# Quantitative analysis of vitreous changes in intraocular inflammation following faricimab treatment in a real world setting

**DOI:** 10.1186/s40942-025-00754-6

**Published:** 2025-12-02

**Authors:** Clemens Thürridl, Konrad Dörfler, Jan Rothbächer, Markus Eidherr, Haidar Khalil, Josef Huemer, Matthias Bolz

**Affiliations:** 1 Department of Ophthalmology, Kepler University Hospital, Linz, Austria; 2 Johannes Kepler University, Linz, Austria; 3 Moorfields Eye Hospital NHS Foundation Trust, London, United Kingdom; 4 NIHR Biomedical Research Centre, UCL Institute of Ophthalmology, London, United Kingdom

**Keywords:** Vitritis, Intraocular inflammation, Faricimab, Intravitreal injection, OCT

## Abstract

**Background:**

To analyze the incidence, and clinical characteristics of intraocular inflammation (IOI) associated with faricimab in a tertiary center in Austria.

**Methods:**

A retrospective analysis of IOI cases following intravitreal faricimab injections in the Kepler University Hospital Linz, Austria. Clinical data was analyzed from January 2023, until March 10th 2025. Clinical and optical coherence tomography (OCT) findings including vitreous changes via mean vitreous grey value (MVGV) were evaluated.

**Results:**

A total of 7333 intravitreal injections of faricimab for a total of 1137 patients were applied during the observation period. We identified 11 eyes with an IOI after faricimab injection with a mean follow up of 22.5 days (range 3–42 days) which corresponds to an incidence of IOI of 0.15% per injection. Quantitative OCT analysis showed a significant rise in mean vitreous gray value during IOI (*p* = 0.002), which returned to baseline after treatment (*p* = 0.63). A significant, fully reversible increase in vitreous haze during IOI episodes was shown. Clinical findings included both anterior and posterior segment inflammation in the absence of pain or occlusive vasculitis.

**Conclusion:**

The results suggest a low incidence of IOI after faricimab injections, comparable to registration trials. MVGV analysis via OCT proved to be sensitive and reproducible. All cases resolved with topical treatment. Intravitreal therapy with faricimab has shown to have a low risk of adverse events.

## Background

Intravitreal injection of anti-vascular endothelial growth factor (VEGF) agents is the standard of care for neovascular age-related macular degeneration (nAMD), diabetic macular edema (DME), and retinal vein occlusions (RVO). Faricimab is a licensed bispecific monoclonal antibody that targets both VEGF-A and Angiopoietin-2 (Ang-2), licensed by the FDA and EMA in 2022 following the results of the phase 3 clinical trials TENAYA, LUCERNE for nAMD, YOSEMITE and RHINE for DME and later, CAMINO and BALATON for RVO [1–3]. 

Faricimab demonstrated a safety profile comparable to Aflibercept 2 mg in the registration trials. Recent real-world studies have provided further insights. A French monocentric study from 2025 reported an overall intraocular inflammation (IOI) incidence of 0.87% per injection and a distinctive presentation of dense peripheral grayish vitritis in 0.63% of cases [4]. Meanwhile, a large-scale analysis from Moorfields Eye Hospital in the UK from 2025 documented a lower overall IOI incidence. Published results range from 0.19% incidence of IOI per injection, to 0.86% per eye, and 0.86% per patient [5]. 

Research shows that optical coherence tomography (OCT) -based signal intensity analysis can correlate well with clinical inflammation scores, offering an alternate method for evaluation of intraocular inflammation, especially in cases with subtle or early inflammatory changes [6]. The use of mean vitreous gray value (MVGV), from standard OCT scans, gives clinicians a relevant tool to evaluate posterior segment inflammation. Building on previous work that validated OCT-based vitreous intensity as a surrogate for inflammation, this study applies MVGV analysis to a real-world cohort of patients treated with faricimab, aiming to describe and quantify IOI episodes with greater precision [7].

## Methods

### Study design and setting

This study was conducted as a retrospective, monocentric review at the Department of Ophthalmology and Optometry at the Kepler University Hospital in Linz, Austria, aiming to analyze all cases of IOI following intravitreal injections of faricimab administered in routine clinical care. The study was approved by the institutional ethics review board of the Johannes Kepler University (EK Nr: 1131/2025) and adhered to the principles of the Declaration of Helsinki. No informed consent was required, since it is retrospective and has a pseudonymized data structure.

All patients who received at least one intravitreal injection of faricimab between January 1, 2023, and March 10, 2025, were included. Data of patients with established diagnoses were further investigated for demographic details. Clinical examination included documentation of anterior segment examination, best-corrected visual acuity (BCVA), and OCT. All OCT scans were made with *“Heidelberg Engineering Spectralis HRA + OCT”* and analysed with *“Heyex Version 2.6.4”*. Cases were excluded if patients were lost to follow-up, essential clinical data were missing or diagnosis was incomplete.

All intravitreal faricimab injections were given according to a set protocol in an established clinical facility. Injections were carried out by resident ophthalmologists in a sterile environment, following the latest guidelines for intravitreal treatment. Injections took place in a designated room designed and equipped for minor ophthalmic procedures, meeting both institutional hygiene standards and national safety rules. All syringes used for intravitreal injections in our department are non-prefilled and are prepared by our hospital pharmacy, using “Zero Residual™” products by “SJJ Solutions” and following “good manufacturing practice” (GMP) - certified standardized protocols.

Clinical data were extracted from the hospital-intern electronic medical records and included patient demographics, ocular diagnosis, number and date of faricimab injections, as well as time of symptom onset, presenting symptoms, anterior and posterior segment findings, and treatment measures. For each IOI case, OCT scans were analyzed at the following three timepoints: before IOI onset, during active IOI, and after resolution. These three timepoints were defined as the day of faricimab injection, day of presentation in our department and first follow-up without signs of inflammation, respectively. Essential demographic data were collected, cases with a history of prior intravitreal injection of another agent at any time were categorized as pre-treated.

For analysis of vitreous inflammation, we used OCT scans with a pre-defined rectangular region of interest (ROI). This ROI was placed in the vitreous above the fovea on the central B-scan. Quantitative OCT analysis of vitreous haze was performed by measuring the mean vitreous gray value within this standardized ROI. If image resolution or export format varied, ROI area was recorded and checked for consistency. MVGV was measured with *“FIJI/ImageJ (version 2.16.0/1.54p)”* [7, 8]. All data were pseudonymized prior to analysis and stored on an encrypted institutional server accessible only to the study team. To compare changes of MVGV between eyes with and without IOI, a control group for OCT analysis was included. The control group was matched for demographics, diagnoses and treated by the same faricimab injection-protocol as the IOI-group. Two timepoints were chosen for MVGV analysis, one before faricimab injection, one after injection.

To minimize potential confounding from media opacity and background signal variability, we applied strict quality-control criteria to all OCT scans. Eyes with dense cataract or other obvious media opacities such as vitreous hemorrhage were excluded. ROI placement was standardized across patients and timepoints. These procedures were oriented on validated protocols for OCT-based vitreous signal quantification described by Keane et al. and Montesano et al. [6, 9]

IOI was defined as the presence of anterior chamber cells, vitreous haze, or other signs of inflammation, in the absence of an infectious causative agent. Diagnostic criteria were based on the Standardization of Uveitis Nomenclature (SUN) classification [10]. Cases of infectious endophthalmitis were excluded.

In this study, “vitritis” refers to clinically visible inflammation of the vitreous body, characterized by vitreous haze and/or the presence of inflammatory cells observed on slit-lamp examination or funduscopy. “Vitreous haze” describes any qualitative or quantitative increase in the optical density of the vitreous on clinical or imaging assessment. “Inflammatory cells” denote discrete hyperreflective dots in the vitreous on clinical examination or OCT scans and were assessed as part of the overall vitreous haze. For quantitative analysis, changes were measured objectively as the mean vitreous gray value within a standardized region of interest above the fovea on OCT B-scans.

The primary outcome was the incidence of intraocular inflammation per faricimab injection. Secondary outcomes included the change in quantitative assessment of MVGV, changes in best-corrected visual acuity, the time from injection to IOI onset, clinical presentation, and treatment response. Visual acuity (logMAR) was documented at three time points: prior to IOI onset, during active IOI, and after inflammation resolution.

All data was extracted using Microsoft Excel spreadsheet. Descriptive statistics were used to summarize demographic and clinical data. Continuous variables were expressed as mean ± standard deviation or median with interquartile range, as appropriate. Categorical variables were described as counts and percentages. For intra-individual comparisons of repeated measurements, the Wilcoxon signed-rank test was applied for paired, nonparametric data. A *p*-value < 0.05 was considered statistically significant.

## Results

During the study period from January 1, 2023, to March 10, 2025, a total of 7333 intravitreal faricimab injections were administered to a total of 1137 patients at the Kepler University Hospital Linz. Of these, 3744 injections were applied to left eyes and 3589 to right eyes. Cases were categorized by diagnosis, showing 580 patients (705 eyes) with nAMD (51%), 248 patients (371 eyes) with DME (21,8%), 163 patients (168 eyes) with RVO (14,3%) and 146 patients (151 eyes) with other diagnoses (12,8%). The group of other diagnoses included choroidal neovascularization (CNV) secondary to central serous chorioretinopathy (CSCR) (*n* = 83), myopic CNV (*n* = 46), Irvine Gass (*n* = 13) and Mb. Coats (*n* = 4). In total, 234 (20.58%) of the 1137 patients were treatment naive, and 903 patients (79.42%) were pre-treated. Diagnoses of those pre-treated patients were nAMD (*n* = 501), DME (*n* = 217), RVO (*n* = 147) and other patients (*n* = 38).

Demographic details of the study cohort are shown in Table 1.

Overall, 11 eyes of 11 patients developed intraocular inflammation, which equates to an overall incidence rate of 0.15% per injection. Of the 11 patients, 5 were male (45.5%) and 6 were female (54.5%). Nine of the 11 IOI cases were pre-treated patients and 2 were treatment naive. Diagnoses of the pre-treated patients were RVO (*n* = 1), nAMD (*n* = 4) and DME (*n* = 4). All pre-treated patients received aflibercept injections prior to faricimab with the last aflibercept injection prior to IOI at a median of 11 months (± 16.82). Pre-treated patients received a median of 8 injections (± 4.30) prior to IOI. Data shown in Table 2.

All 11 IOI cases experienced painless visual disturbances, typically described as either blurred vision or floaters. The initial symptoms occurred between 3 and 42 days following the most recent faricimab injection. None of the affected patients developed systemic symptoms or signs suggestive of an infectious etiology. Ophthalmic findings showed vitreous haze, anterior segment inflammation ranging from SUN 0.5 + to 3+, with presence of inflammatory cells in the vitreous. The intraocular pressure remained within normal limits in all affected eyes. Of the 11 cases of IOI eight were pseudophakic, two had lens opacity graded as mild nuclear cataract (NC) and one case showed posterior capsular opacification (PCO), in the same eye as IOI, respectively. Features and findings of all 11 cases are shown in Table 3.

**Table 1:** Study cohort

Diagnosis	Patients (%)	M/F (%)	Naive/Pre-treated	Age (Median ± SD)	Total Eyes	Eyes (*R*/L)
*nAMD*	*580 (51)*	*231/349 (39*,*9/60*,*1)*	*79/501*	*82.0 ± 8.8*	*705*	*337/368*
*DME*	*248 (21*,*8)*	*160/88 (64*,*5/35*,*5)*	*31/217*	*69.0 ± 11.9*	*371*	*193/178*
*RVO*	*163 (14*,*3)*	*87/76* *(53*,*4/46*,*6)*	*16/147*	*75.0 ± 11.1*	*168*	*85/83*
*Others*	*146 (12*,*8)*	*73/73* *(50/50)*	*108/38*	*66.8 ± 15.1*	*151*	*68/83*
*Total*	*1137*	*551/586 (48*,*5/51*,*5)*	*234/903*	*73*,*2 ± 11*,*7*	*1395*	*683/712*

**Table 2:** Prior treatment status of IOI cases

	naive	pre-treated	total
nAMD	2	4	6
DME	0	4	4
RVO	0	1	1
median prior injections (± SD)	3 (2.83)	8 (4.32)	8 (4.32)
median months between prior treatment and IOI (± SD)	-	11 (± 16.82)	-

**Table 3:** Findings of the 11 IOI cases

Case	age	sex	Diagnosis	Prior faricimab injections (#)	Anterior segment presentation (SUN)	Lens	before	MVGV during	after
1	58	m	RVO	9	2+	pseudophakic	4,55	21,52	4,96
2	77	f	nAMD	1	1+	pseudophakic	8,37	22,07	11,57
3	79	f	nAMD	2	3+	pseudophakic	7,29	43,43	4,56
4	86	f	nAMD	16	3+	pseudophakic	5,45	83,93	7,96
5	78	m	DME	9	2+	pseudophakic	12,02	21,70	7,67
6	75	m	nAMD	3	0.5+	Mild NC	4,69	12,66	6,02
7	67	m	DME	8	2+	Mild NC	14,72	16,40	16,20
8	89	f	nAMD	3	1+	PCO	10,85	18,46	2,89
9	57	m	DME	9	0.5+	pseudophakic	6,34	18,77	13,59
10	57	m	DME	8	1+	pseudophakic	13,01	33,48	16,23
11	79	f	nAMD	5	1+	pseudophakic	14,80	27,30	25,93

### Optical coherence tomography findings

**Fig. 1:**
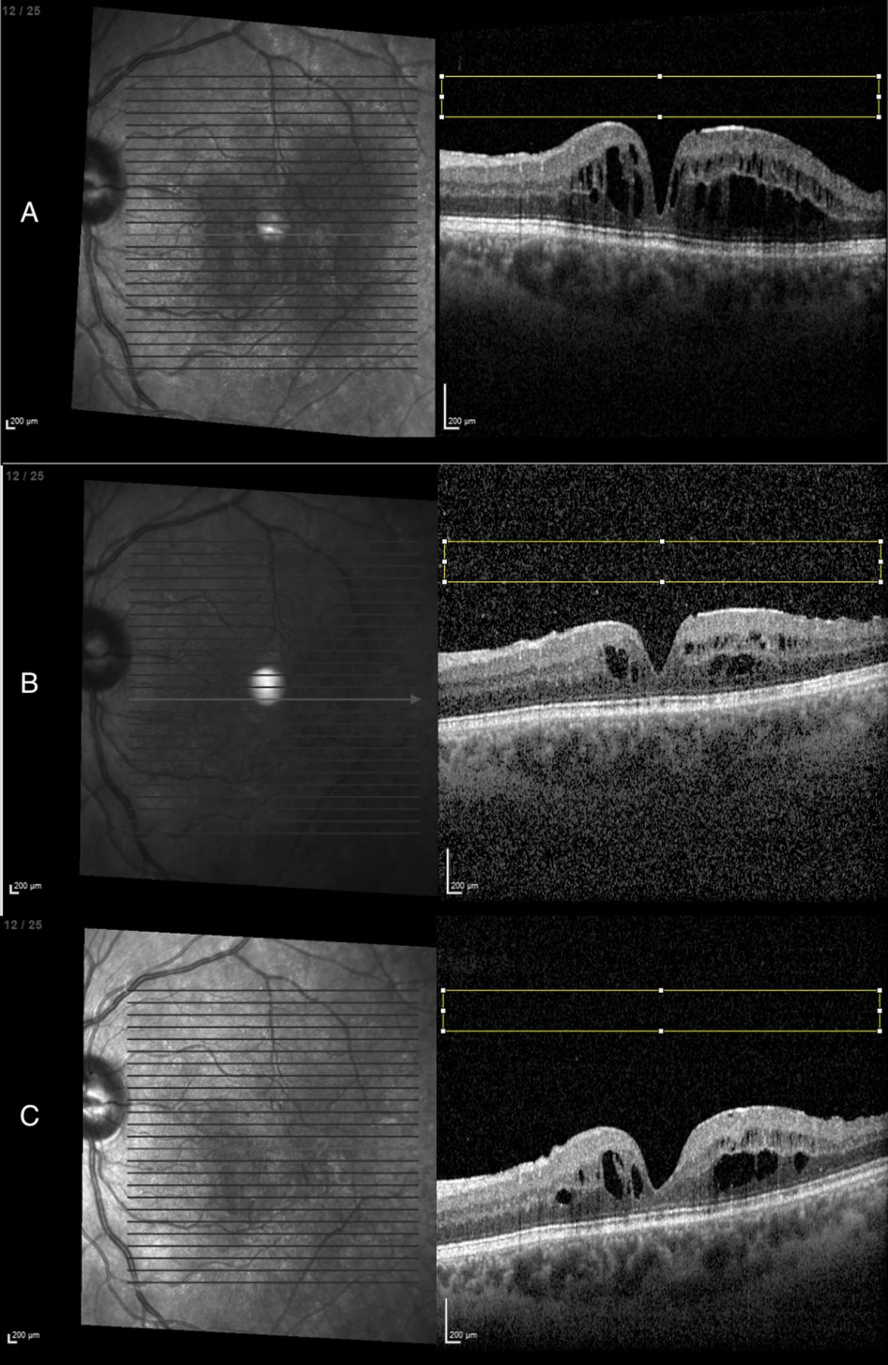
Example of mean vitreous gray value changes of one patient with intraocular inflammation after intravitreal faricimab injection. **A** before IOI, **B** during IOI, **C** after IOI. Rectangle in yellow as defined region of interest (ROI)

OCT imaging revealed variable degrees of intra- and subretinal fluid in affected eyes at baseline. Post-injection OCTs performed at the time of IOI diagnosis demonstrated a marked increase in vitreous signal density and shadowing, consistent with vitreous haze. Quantitative evaluation demonstrated a significant increase in mean vitreous gray value during IOI compared to baseline (median before IOI: 9.6, during IOI: 21.9, after IOI: 7.8; Wilcoxon signed-rank test, V = 0, *p* = 0.002). After treatment, mean vitreous intensity declined significantly (V = 55, *p* = 0.002) and returned to values not significantly different from baseline (V = 22, *p* = 0.63). For the control group, median MVGV of the two measurements were 9.34 (before) and 7.85 (after) (Wilcoxon signed-rank test, V = 21, *p* = 0.32). An example of OCT MVGC changes of one IOI patient is presented in Fig. 1.

### Treatment and clinical outcomes

All cases of IOI were managed with topical corticosteroid therapy (Prednisolon acetat 1%, Agepha pharma) in a tapering regimen as by doctors’ discretion. No cases requiring systemic corticosteroids were noted. Inflammation completely resolved in all eyes within 1 to 4 weeks after treatment initiation.

A significant decline in visual acuity occurred during IOI compared to baseline (V = 0, *p* = 0.0025, Wilcoxon signed-rank test). Visual function improved significantly after IOI resolution (V = 78, *p* = 0.0024). The baseline and post-IOI values showed no significant difference (V = 19, *p* = 0.944) which indicated complete recovery.

None of the eleven cases of IOI showed persistent visual impairment or required a change in therapeutic strategy. 

## Discussion

In this retrospective analysis of 7,333 intravitreal faricimab injections, we observed 11 cases of sterile intraocular inflammation, resulting in an overall incidence of 0.15% per injection, with all cases returning to a baseline MVGV after IOI resolution. All IOI cases were characterized by mild to moderate vitreous haze, without anterior chamber involvement, vasculitis, or signs of infection. Furthermore, all cases responded well to topical corticosteroid therapy and none of the patients suffered long-term visual loss.

OCT-based analysis of vitreal haze in uveitic conditions, as shown by Liu et al., provides a quantitative measurement of inflammatory activity of the posterior hyaloid. A significant association between vitreous intensity and visual acuity was displayed in a group of 119 patients [11]. While they examined OCT scans at one timepoint, we examined the course of inflammation over three different timepoints. In our case, OCT monitoring of MVGV proved valuable for evaluating IOI in our patients and documenting the course and resolution of the disease. Therefore, our study provides objective evidence of vitreous inflammation by quantifying MVGV on OCT scans. We observed a significant increase in vitreous signal intensity during IOI episodes, as measured by a standardized region of interest above the fovea, with values returning to baseline in all cases after resolution of IOI.

The vitreous OCT-derived signal’s specific nature as an inflammatory marker remains under investigation because media opacity and background noise can modify gray-level intensity. Our study used precise scan-quality evaluation together with consistent ROI positioning to minimize the impact of these variables. The analysis of our study demonstrates that OCT-based vitreous signal quantification remains precise even when these potential confounding elements are present. After adjusting for phakic status and previous vitrectomy, Zarranz-Ventura et al. found that OCT vitreous intensity results closely match clinical vitreous haze measurements because relative signal measurements remain stable despite media opacity. According to Sreekantam et al., vitreous signal intensity decreases with anti-inflammatory treatment while showing correlation with functional improvement. OCT signal strength relies on specific acquisition settings, but standardized scanning methods yield dependable results for medical use [9, 12, 13]. The data confirms the pattern of MVGV levels showing an increase during active IOI episodes which later return to normal values after resolution.

Our approach provides a sensitive and reproducible way of monitoring posterior segment inflammation, complementing conventional grading and proved to be a clinically relevant tool. This is consistent with the complete reversibility of faricimab-associated IOI observed in our cohort. These findings are further in line with reports highlighting the utility of OCT-based quantification of vitreous haze as an additional biomarker in inflammatory ocular disease [7]. Prior works on OCT-based vitreous analysis found in literature support this approach as a meaningful biomarker. Keane et al. measured vitreous intensity relative to retinal pigment epithelium intensity on OCT significantly correlated with clinical vitreous haze scores, offering an objective and reproducible alternative to slit lamp examination, which can suffer from examiner bias [6]. We suspect ever more incorporation of optimized vitreous analysis in inflammatory diseases of the eye, as well as AI-based vitreous analysis, similar to already used AI-systems, some of them even in a semi-automated fashion [9, 11, 14]. 

Our incidence rate of IOI aligns with previous real-world reports, showing generally low rates. A French single-center investigation by Bourdin et al. documented an IOI rate of 0.87% with vitritis in 0.63% of 1,271 faricimab injections. Their cases were characterized by dense, peripheral vitreous inflammation and in some instances required systemic corticosteroids [4]. The IOI occurrence rate from our study was six times lower and only topical therapy was needed for treatment. A Moorfields Eye Hospital study from the United Kingdom found that the IOI incidence per faricimab injection reached 0.19% after analyzing more than 10,000 doses [5]. This study showed that 20% of all IOI cases appeared as vitritis. There were no cases of posterior vasculitis or visual loss. Our findings demonstrate a similar rate of IOI occurrences but featured less severe symptoms, without the need of systemic therapy, with vitreous haze being the most common IOI manifestation. Of interest, the median number of injections prior to IOI in our cohort was higher than other studies with a median of 8 injections (range 1–16) or a mean of 6.5 (± 4.3) injections. Meanwhile Bourdin et al. described a mean number of 3.0 (± 1.5) injections prior to IOI and Montesel et al. reported a median of 3.5 injections (range 1–10) [4, 5]. 

The TRUCKEE study by Khanani et al. delivers additional evidence that faricimab injections lead to very low rates of IOI. The study observed 376 eyes and recorded only one IOI case which equates to an incidence rate of 0.27%. The short duration of three months in follow-up restricts the ability to determine the occurrence of inflammation afterwards, plus the observed amount of eyes is low [15]. 

Different rates of IOI observed in various medical centers may result from variations in medical observation methods and patient definitions as well as patient profiles and injection protocols. Our population did not experience any cases of infectious endophthalmitis or occlusive vasculitis or permanent retinal damage which demonstrates the safety of faricimab under structured clinical treatment.

The main limitation of our research is its retrospective, single-center nature which may reduce the applicability of the results to broader populations. As with all retrospective studies, mild or subclinical inflammation can be missed due to underreporting. Additionally, the absence of a control group with other intravitreal injections (e.g., aflibercept or ranibizumab) rules out a direct comparison of IOI risk between different agents. Our dataset doesn’t contain detailed systemic or laboratory inflammatory markers, and aqueous or vitreous samples weren’t taken or analyzed. Hence, while every case was clinically consistent with sterile inflammation, subclinical infection can not be entirely excluded. Lastly, the short follow-up period after IOI resolution limits assessment of possible delayed recurrences. Despite minor differences in OCT export format and ROI area, the consistency of measurement reliability remained consistent. Additional prospective research should validate the results through controlled imaging protocols, as this study did not eliminate all optical differences, such as faint lens clouding and posterior capsule effects.

## Conclusion

Though mentioned limitations are present, our study represents one of the largest real-world evaluations of IOI following faricimab in Central Europe up to date and one of few works that addresses changes of vitreous during faricimab induced IOI, measured by OCT. Our retrospective, single-center study from Austria confirmed that sterile intraocular inflammation following intravitreal faricimab injections is a rare but clinically relevant complication, with an incidence of 0.15% per injection.

## Data Availability

The datasets generated and analysed during the current study are not publicly available due to their location on local servers of the Department of Ophthalmology, Kepler University Hospital, Linz, Austria, but are available from the corresponding author on reasonable request.

## Declarations

### Generative AI and AI-assisted technologies in the writing process

After completing the writing and editing of the manuscript, the authors used ChatGPT exclusively for the final revision and minor adjustments. Following the use of this tool, the authors reviewed and edited the content as needed and take full responsibility for the content of the published article

## Abbreviations

BCVA: Best-corrected visual acuity
CSCR: Central serous chorioretinopathy
CNV: Choroidal neovascularization
DME: Diabetic macular edema
IOI: Introcular inflammation
logMAR: Logarithm of the minimum angle of resolution
MVGV: Mean vitreous grey value
nAMD: Neovascular age-related macular degeneration
NC: Nuclear cataract
OCT: Optical coherence tomography
PCO: Posterior capsular opacification
ROI: Region of interest
RVO: Retinal vein occlusion
SUN: Standardization of Uveitis Nomenclature
VEGF: Vascular endothelial growth factor
